# Supplementary material for: Estimation of energy efficiency of heat pumps in residential buildings using real operation data
Source: Nat Commun. 2025 Mar 22;16:2834. doi: 10.1038/s41467-025-58014-y (PMC11929890; doi:10.1038/s41467-025-58014-y)
Supplement: Supplementary file 1 — Supplementary Information [file 41467_2025_58014_MOESM1_ESM.pdf]

Supplementary Information:

Estimation of energy efficiency of heat pumps in residential buildings using real operation data

Tobias Brudermueller[1*], Ugne Potthoff[1], Elgar Fleisch[1,2], Felix Wortmann[2*], Thorsten Staake[1,3]

[1*]Chair of Information Management, ETH Zurich, Weinbergstrasse 56/58, Zurich, 8092, Switzerland.

[2]Institute of Technology Management, University of St. Gallen, Dufourstrasse 40a, St. Gallen, 9000, Switzerland.

[3]Chair of Information Systems and Energy Efficient Systems, University of Bamberg, An der Weberei 5, Bamberg, 96047, Germany.

*Corresponding author(s). E-mail(s): tbrudermuell@ethz.ch; felix.wortmann@unisg.ch;

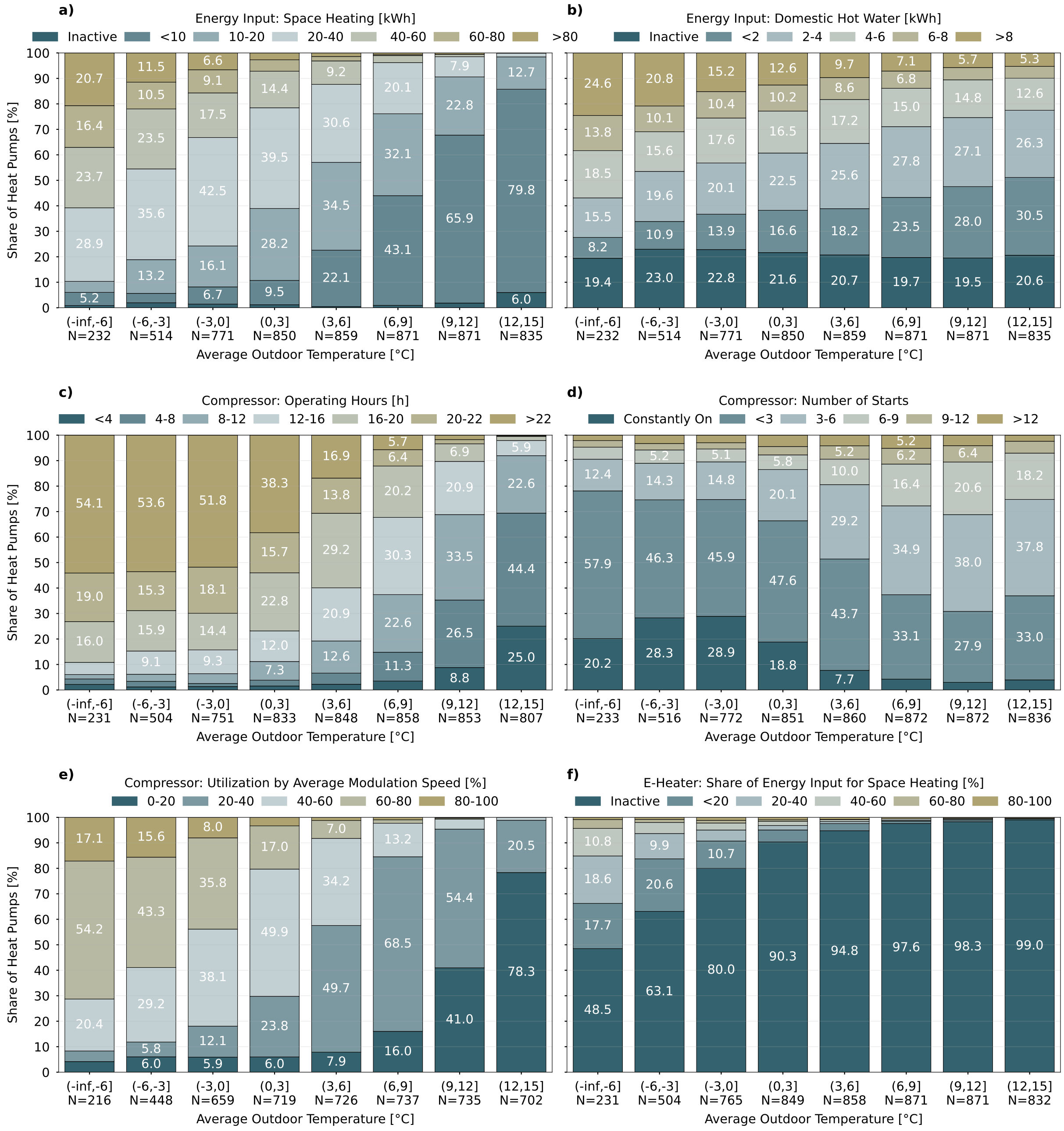

Supplementary Figure 1 Additional performance indicators of the individual air-source heat pumps studied. A single vertical bar represents a histogram of median values calculated for individual air-source heat pumps within specific temperature ranges and different operating modes. The y-axes are shared within each row. (a) Energy input (in kWh) in space heating mode. (b) Energy input (in kWh) in domestic hot water mode. (c) Operating hours of the compressor. (d) Number of compressor starts. (e) Average compressor modulation (in %). (f) Share of energy input of the electric heater (in %) in space heating mode. Source data are provided as a Source Data file.

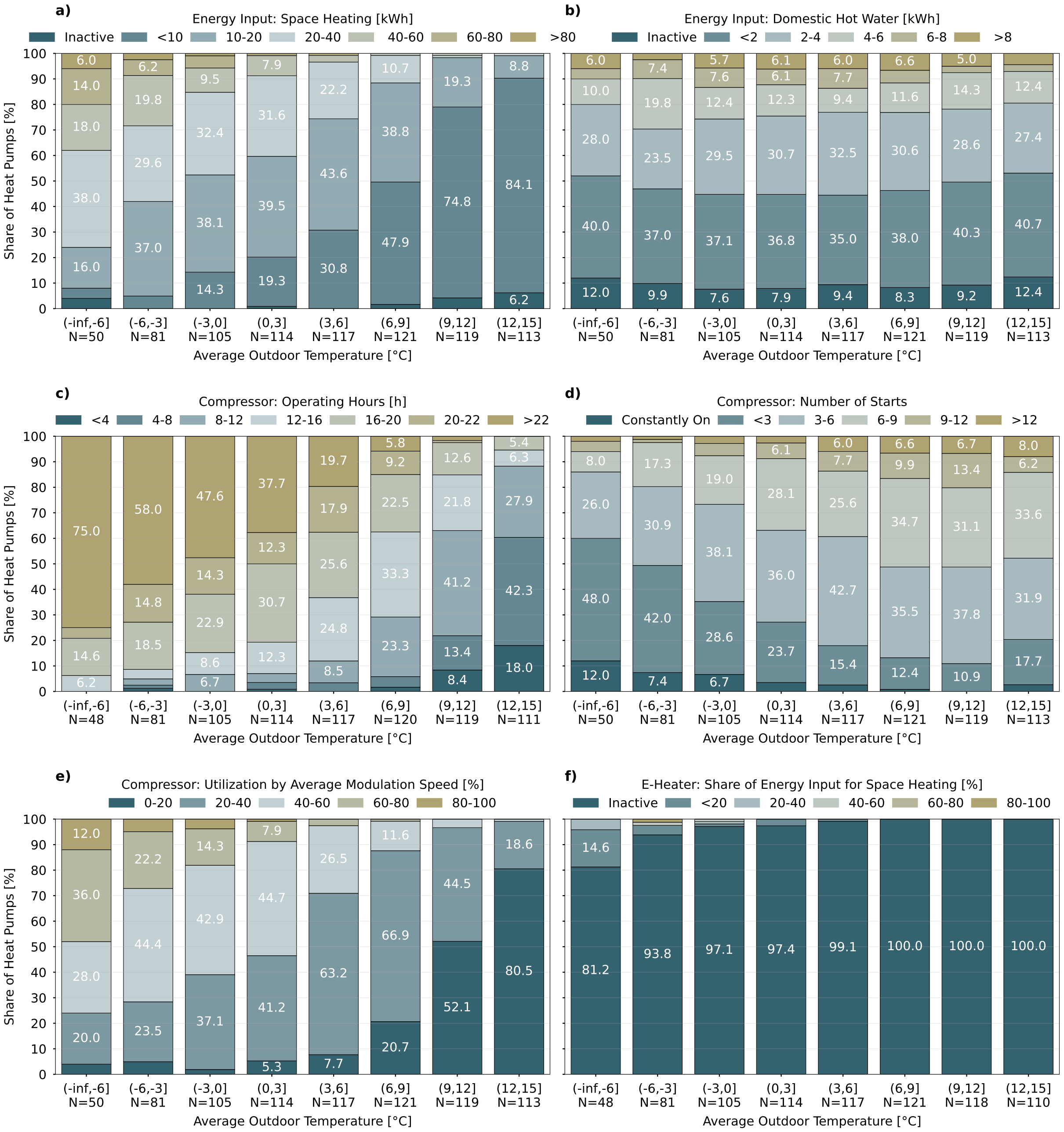

Supplementary Figure 2 Additional performance indicators of the individual ground-source heat pumps studied. A single vertical bar represents a histogram of median values calculated for individual ground-source heat pumps within specific temperature ranges and different operating modes. The y-axes are shared within each row. (a) Energy input (in kWh) in space heating mode. (b) Energy input (in kWh) in domestic hot water mode. (c) Operating hours of the compressor. (d) Number of compressor starts. (e) Average compressor modulation (in %). (f) Share of energy input of the electric heater (in %) in space heating mode. Source data are provided as a Source Data file.

Supplementary Table 1 Fixed test points are established for medium and low-temperature applications across various climate zones as defined in EN 14825. However, the standard does not specify an average temperature application; we have defined it ourselves as the average of the low and medium temperature applications. For completeness, the defined part-load ratios are also provided.

Climate	HP Type	Temperature Application	Outdoor Temperatures [°C]	Weights	Part Load Ratios [%]	Supply Temperatures [°C]
Average	ASHP	Low<br>Medium<br>Average	{-7, 2, 7, 12}	{24, 320, 326, 169}	{88, 54, 35, 15}	{34, 30, 27, 24}<br>{52, 42, 36, 30}<br>{43, 36, 31.5, 27}
	GSHP	Low<br>Medium<br>Average	{0, 0, 0, 0}	{24, 320, 326, 169}	{88, 54, 35, 15}	{34, 30, 27, 24}<br>{52, 42, 36, 30}<br>{43, 36, 31.5, 27}
Warm	ASHP	Low<br>Medium<br>Average	{2, 7, 12}	{3, 162, 503}	{100, 64, 29}	{35, 31, 26}<br>{55, 46, 34}<br>{45, 38.5, 30}
	GSHP	Low<br>Medium<br>Average	{0, 0, 0}	{3, 162, 503}	{100, 64, 29}	{35, 31, 26}<br>{55, 46, 34}<br>{45, 38.5, 30}
Cold	ASHP	Low<br>Medium<br>Average	{-15, -7, 2, 7, 12}	{41, 125, 380, 269, 146}	{82, 61, 37, 24, 11}	{32, 30, 27, 25, 24}<br>{49, 44, 37, 32, 28}<br>{40.5, 37, 32, 28.5, 26}
	GSHP	Low<br>Medium<br>Average	{0, 0, 0, 0, 0}	{41, 125, 380, 269, 146}	{82, 61, 37, 24, 11}	{32, 30, 27, 25, 24}<br>{49, 44, 37, 32, 28}<br>{40.5, 37, 32, 28.5, 26}

Supplementary Table 2 Performance thresholds derived from European regulations for certifying and labeling heat pump products under controlled laboratory conditions. Closed brackets denote that the value is included in the interval, while open brackets indicate that the value is excluded. Thresholds are provided for seasonal space heating energy efficiency (SSHEE) and seasonal coefficient of performance (SCOP) for both air-source heat pumps (ASHPs) and ground-source heat pumps (GSHPs).

Category	Low Temperature Application			Medium Temperature Application			Average of Low and Medium Temperature Application		
	SSHEE (All)	SCOP (GSHP)	SCOP (ASHP)	SSHEE (All)	SCOP (GSHP)	SCOP (ASHP)	SSHEE (All)	SCOP (GSHP)	SCOP (ASHP)
Opt. Optional	[125, ∞)	[3.33, ∞)	[3.20, ∞)	[110, ∞)	[2.95, ∞)	[2.83, ∞)	[117.5, ∞)	[3.14, ∞)	[3.01, ∞)
Opt. Required	[0, 125)	[0.00, 3.33)	[0.00, 3.20)	[0, 110)	[0.00, 2.95)	[0.00, 2.83)	[0.0, 117.5)	[0.00, 3.14)	[0.00, 3.01)
A+++	[175, ∞)	[4.58, ∞)	[4.45, ∞)	[150, ∞)	[3.95, ∞)	[3.83, ∞)	[162.5, ∞)	[4.26, ∞)	[4.14, ∞)
A++	[150, 175)	[3.95, 4.58)	[3.83, 4.45)	[125, 150)	[3.33, 3.95)	[3.20, 3.83)	[137.5, 162.5)	[3.64, 4.26)	[3.51, 4.14)
A+	[123, 150)	[3.26, 3.95)	[3.15, 3.83)	[98, 125)	[2.65, 3.33)	[2.53, 3.20)	[110.5, 137.5)	[2.96, 3.64)	[2.84, 3.51)
A	[115, 123)	[3.08, 3.26)	[2.95, 3.15)	[90, 98)	[2.45, 2.65)	[2.33, 2.53)	[102.5, 110.5)	[2.76, 2.96)	[2.64, 2.84)
B	[107, 115)	[2.88, 3.08)	[2.75, 2.95)	[82, 90)	[2.25, 2.45)	[2.13, 2.33)	[94.5, 102.5)	[2.56, 2.76)	[2.44, 2.64)
C	[100, 107)	[2.70, 2.88)	[2.58, 2.75)	[75, 82)	[2.08, 2.25)	[1.95, 2.13)	[87.5, 94.5)	[2.39, 2.56)	[2.26, 2.44)
D	[61, 100)	[1.73, 2.70)	[1.60, 2.58)	[36, 75)	[1.10, 2.08)	[0.98, 1.95)	[48.5, 87.5)	[1.41, 2.39)	[1.29, 2.26)
E	[59, 61)	[1.68, 1.73)	[1.55, 1.60)	[34, 36)	[1.05, 1.10)	[0.93, 0.98)	[46.5, 48.5)	[1.36, 1.41)	[1.24, 1.29)
F	[55, 59)	[1.58, 1.68)	[1.45, 1.55)	[30, 34)	[0.95, 1.05)	[0.83, 0.93)	[42.5, 46.5)	[1.26, 1.36)	[1.14, 1.24)
G	[0, 55)	[0, 1.58)	[0, 1.45)	[0, 30)	[0.00, 0.95)	[0.00, 0.83)	[0.0, 42.5)	[0.00, 1.26)	[0.00, 1.14)

Supplementary Note 1: Testing models for robustness

In the main article, we introduce models for the heating curve, COP, and utilization. All these models have been fitted using the entire data set, ensuring that the performance assessment of each HP is based on all available observations.

To further assess the robustness of our models, we also evaluated their performance by splitting the data into 70% for training and 30% for testing, and then comparing the results. This data split was performed individually for each HP by randomly selecting from the daily observations, ensuring that each system is represented in both the training and test sets. We chose this approach over cross-validation to strike a balanced compromise. In a cross-validation approach, it would have been necessary to calculate the average scores for each HP across all folds and then aggregate these averages, which could have limited the interpretability of the presented scores. Further note that, for consistency, the same 708 HPs are used for evaluating both the heating curve and COP models, while the same 637 HPs are used to evaluate the utilization model, in alignment with the rest of this paper.

Supplementary Table 3 presents the mean and standard deviations of the scores for models fitted exclusively to the training data, evaluated separately on both the training and test data sets. For comparison, the table also includes the scores from the models used throughout this paper, which were fitted to the entire data set (see the rows indicating that all data was used). These rows accurately reproduce the scores presented in Table 1 of the main article. The results show that the models are able to generalize well, as the difference in error between the training and test sets is minimal. Similarly, the difference in scores between the model fitted on the entire data set and those using the data split is also negligible. This supports our decision to use the models fitted on the entire data set, as they provide better interpretability by incorporating all observations into the performance assessment.

Supplementary Table 3 Mean and standard deviation (in brackets) of model fit scores when randomly splitting the data of each heat pump into 70% for training and 30% for testing. These results are also compared to the models used throughout the analyses in this paper, as presented in Table 1 of the main article.

Model Type	Data	MdAE	MAE	MSE	RMSE	MAPE	SMAPE	R^2
Heating Curve	All	1.02 (0.69)	1.20 (0.75)	2.99 (4.58)	1.48 (0.90)	3.32 (2.06)	3.30 (2.02)	0.43 (0.27)
	Train	1.03 (0.69)	1.19 (0.75)	2.98 (4.55)	1.48 (0.9)	3.31 (0.02)	3.29 (2.01)	0.43 (0.27)
	Test	1.05 (0.76)	1.22 (0.79)	3.12 (4.98)	1.5 (0.93)	3.39 (0.02)	3.37 (2.14)	0.30 (1.31)
COP	All	0.19 (0.11)	0.24 (0.13)	0.12 (0.14)	0.30 (0.18)	5.65 (2.48)	5.58 (2.38)	0.75 (0.17)
	Train	0.19 (0.11)	0.23 (0.13)	0.12 (0.14)	0.30 (0.17)	5.64 (0.03)	5.57 (2.40)	0.75 (0.17)
	Test	0.20 (0.12)	0.24 (0.14)	0.13 (0.16)	0.31 (0.18)	5.79 (0.03)	5.73 (2.53)	0.72 (0.23)
Utilization	All	4.66 (1.87)	5.59 (2.11)	57.48 (52.00)	7.11 (2.64)	21.18 (0.13)	17.49 (7.06)	0.65 (0.19)
	Train	4.68 (1.94)	5.59 (2.14)	57.62 (52.48)	7.10 (2.68)	21.20 (0.13)	17.44 (7.13)	0.64 (0.21)
	Test	4.77 (2.08)	5.69 (2.22)	58.97 (56.17)	7.17 (2.75)	21.78 (0.15)	17.90 (7.60)	0.60 (0.41)

Supplementary Note 2: Comparison to heat pump performance in the literature

As mentioned in the introduction of the main article, the number of HP field studies that extend beyond single system analysis is quite limited. A series of well-known in-situ measurement projects conducted in Germany by the Fraunhofer Institute includes WP-Effizienz [1] (2005-2010, Germany, 18 ASHPs, 56 GSHPs), WP-Monitor [2] (2009-2013, Germany, 35 ASHPs, 47 GSHPs), and WP-Smart [3] (2015-2018, Germany, 32 ASHPs, 13 GSHPs), with their findings summarized by the authors in [4]. The first two projects focus on newly constructed buildings, whereas the last project specifically addresses older buildings. Equally notable is the SEPEMO project [5] (2009-2012, several European countries, 9 ASHPs), which was the first to establish clear system boundaries for evaluating HP performance. Additionally, the UCL-RHPP project [6] (2013-2015, UK, 292 ASHPs, 92 GSHPs) covers 384 installations in the

Supplementary Table 4 Our performance scores compared to figures reported in other field studies. These include seasonal performance factor (SPF) and seasonal coefficient of performance (SCOP) in either space heating (SH) or domestic hot water (DHW) mode of operation.

HP Type	Study	Metric	Operating Modes	N_{HP}	Min	Mean	Std	Max
ASHPs	Ours	$\mathrm{SCOP_{real}}$	SH	612	1.83	3.72	0.71	5.55
	WP-Smart [3]	$\mathrm{SPF_{H3}}$	SH	39	1.50	3.30		5.00
	WP-Smart [3]	$\mathrm{SPF_{H3}}$	SH & DHW	35	2.10	2.60		3.30
	WP-Effizienz [1]	$\mathrm{SPF_{H3}}$	SH & DHW	18	2.30	2.90		3.40
	WP-Monitor [2]	$\mathrm{SPF_{H3}}$	SH & DHW	35	2.20	3.10		4.20
	SEPEMO [5]	$\mathrm{SPF_{H3}}$	SH & DHW	9		3.38	0.50	
	UCL-RHPP [6]	$\mathrm{SPF_{H2}}$	SH	292		2.72		
	UCL-RHPP [6]	$\mathrm{SPF_{H2}}$	SH & DHW	292		2.64		
GSHPs	Ours	$\mathrm{SCOP_{real}}$	SH	96	2.86	4.80	0.86	7.36
	WP-Smart [3]	$\mathrm{SPF_{H3}}$	SH	13	1.80	4.20		5.40
	WP-Smart [3]	$\mathrm{SPF_{H3}}$	SH & DHW	36	2.20	3.30		4.30
	WP-Effizienz [1]	$\mathrm{SPF_{H3}}$	SH & DHW	56	3.10	3.90		5.10
	WP-Monitor [2]	$\mathrm{SPF_{H3}}$	SH & DHW	45	3.00	4.00		5.40
	UCL-RHPP [6]	$\mathrm{SPF_{H2}}$	SH	92		3.03		
	UCL-RHPP [6]	$\mathrm{SPF_{H2}}$	SH & DHW	92		2.93		

UK, making it the largest field study known to us besides our own. The results of these projects, along with those from several smaller studies, are summarized in the study by O'Hegarty et al. [7] and in a meta-analysis by Gleeson and Lowe [8]. Please note that the report for the SEPEMO project is no longer accessible, as it has been archived and removed from the European Commission's projects database.

As discussed by O'Hegarty et al. [7], the performance scores presented in this study can be compared to the $\mathrm{SPF_{H3}}$ metrics from the aforementioned studies. Notably, these scores are absent in the reports of the UCL-RHPP project [6], which exclusively provides values for the H_2 and H_4 system boundaries. For comparisons with other studies, refer to Supplementary Table 4 where we have summarized the results. For the Fraunhofer projects, we rely on the figures reported in the authors' summary [4], noting that the number of HPs analyzed may be fewer than the total number of systems included in the project. From the comparison, it can be concluded that the HPs in our data set generally exhibit higher performance than those in other field studies, although with greater variability. However, our reported scores pertain only to SH, whereas most other studies also include DHW without specifying the number of HPs used for DHW production or their corresponding share. Differences in building types and geographic regions can also contribute to the observed discrepancies. For this reason, we emphasize that the comparison made here should be treated with caution.

In addition to comparing field performance, we also evaluate our COP model (see Equation 4 of the main article) against other models in the literature that use values from product certificates. Note that such models, which account for outdoor temperature dependence, exist only for ASHPs. Therefore, our comparisons are limited to this category. The results are illustrated in Supplementary Figure 3, where we also present the ratio of the mean COP from our model to those of other models, sampled at temperatures between -10 °C and 15 °C. The model proposed by Pospíšil et al. [9] is given by:

$$\mathrm{COP} = 0.0023 \cdot (T_{\mathrm{supp}} - T_{\mathrm{out}})^2 - 0.2851 \cdot (T_{\mathrm{supp}} - T_{\mathrm{out}}) + 10.677 \quad (1)$$

While Lämmle et al. [10] simply illustrates Carnot efficiency with a correction factor $\zeta = 0.5$ (see Equation 1 of the main article), Ruhnau et al. [11] defines a model originally proposed by Fischer et al.

[12], as follows:

$$\text{COP} = 6.08 - 0.0005 \cdot (T_{\text{supp}} - T_{\text{out}})^2 - 0.09 \cdot (T_{\text{supp}} - T_{\text{out}}) \tag{2}$$

The graph in Supplementary Figure 3 shows that the HPs in this study operate between the COP models defined by Pospíšil et al. [9] and Fischer et al. [12], with the observed Carnot correction factor ζ being approximately between 0.25 and 0.33. By providing our models as supplementary material, future studies can utilize them to model COP more realistically, based on real-world measurements rather than product certificates, and including standard deviations.

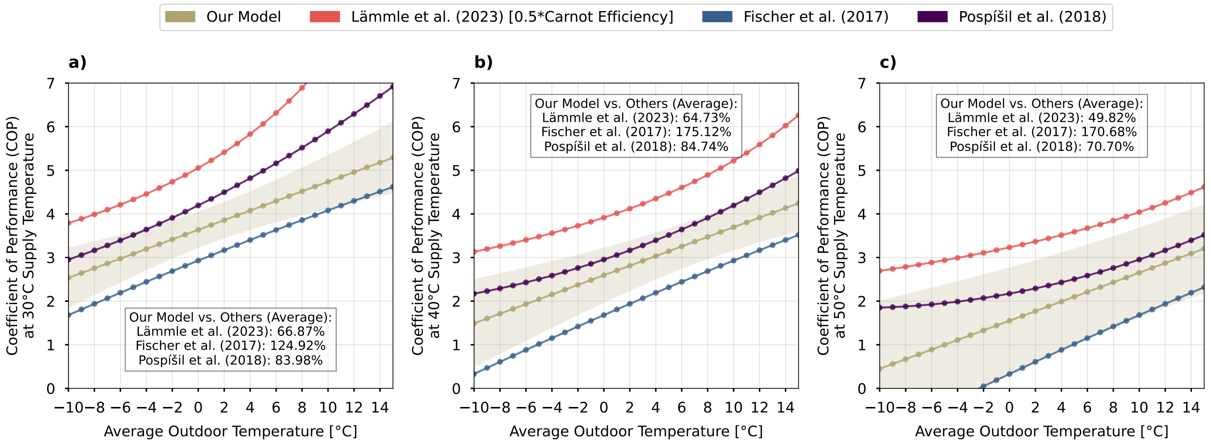

Supplementary Figure 3 Comparison of our coefficient of performance (COP) model for air-source heat pumps to other models in the literature. Note that the other models are based on product certificates rather than real-world measurements. Comparison with fixed supply temperatures of: (a) 30 °C (b) 40 °C (c) 50 °C. Source data are provided as a Source Data file.

Nomenclature

Abbreviations

ASHP	Air-source heat pump
COP	Coefficient of performance
CR	Capacity ratio
DHW	Domestic hot water
GSHP	Ground-source heat pump
HP	Heat pump
IEA	International Energy Agency
MAE	Mean absolute error
MAPE	Mean absolute percentage error
MdAE	Median absolute error
MSE	Mean squared error
PLR	Part-load ratio
RMSE	Root mean square error
SCOP	Seasonal coefficient of performance
SMAPE	Symmetric mean absolute percentage error
SH	Space heating
SPF	Seasonal performance factor
SSHEE	Seasonal space heating energy efficiency
UK	United Kingdom
RHPP	Renewable Heat Premium Payment

Symbols

i	Index of a particular HP
η	Seasonal space heating energy efficiency [%]
ζ	Second law efficiency correction factor for Carnot efficiency
$\text{COP}^i(T_{\text{out}}, T_{\text{supp}})$	Predicted coefficient of performance
d^i_{ASHP}	Dummy variable: 1 if HP indexed i is an ASHP, else 0
d^i_{GSHP}	Dummy variable: 1 if HP indexed i is a GSHP, else 0
ΔE	Difference in electricity consumption [kWh]
E_{new}	Electricity after heating curve reduction [kWh]
E_{old}	Electricity before heating curve reduction [kWh]
$F(1)$	Correction factor: temperature controls [%]
$F(2)$	Correction factor: water-to-water/water-to-air systems [%]
$\text{PLR}(T_j)$	Part-load ratio at a given outdoor temperature
Q_{heat}	Heat demand [kWh]
$\text{SCOP}^i_{\text{real}}$	Predicted seasonal coefficient of performance
T_j	Any specific outdoor temperature [°C]
T_{design}	Design temperature [°C]
T_{hsource}	Heat source temperature [K]
T_{hsupply}	Heat supply temperature [K]
T_{lim}	Heating limit temperature [°C]
T_{out}	Outdoor temperature [°C]
T_{supp}	Supply temperature [°C]
$T^i_{\text{supp}}(T_{\text{out}})$	Predicted supply temperature [°C]
$\text{Utilization}^i(T_{\text{out}})$	Predicted utilization [%]

Supplementary References

[1] Miara, M., Günther, D., Kramer, T., Oltersdorf, T., Wapler, J.: Wärmepumpen Effizienz Messtechnische Untersuchung von Wärmepumpenanlagen zur Analyse und Bewertung der Effizienz im realen Betrieb. Fraunhofer Institut für Solare Energiesysteme (ISE), Freiburg (2011). Accessed: 2024 June 03

[2] Günther, D., Miara, M., Langner, R., Helmling, S., Wapler, J.: "WP Monitor" Feldmessung von Wärmepumpenanlagen. Fraunhofer Institut für Solare Energiesysteme (ISE), Freiburg (2014). Accessed: 2024 June 03

[3] Günther, D., Wapler, J., Langner, R., Helmling, S., Miara, M., Fischer, D., Zimmermann, D., Wolf, T., Wille-Hausmann, B.: Wärmepumpen in Bestandsgebäuden: Ergebnisse aus dem Forschungsprojekt "WPsmart im Bestand". Abschlussbericht 03ET1272A (2020). Accessed: 2024 June 03

[4] Miara, M., Günther, D., Langner, R., Helmling, S., Wapler, J.: 10 years of heat pumps monitoring in Germany. Outcomes of several monitoring campaigns. from low-energy houses to un-retrofitted single-family dwellings. In: 12th IEA Heat Pump Conference, p. 11 (2017)

[5] Nordman, R., Kleefkens, O., Riviere, P., Nowak, T., Zottl, A., Arzano-Daurelle, C., Lehmann, A., Polyzou, O., Karytsas, K., Riederer, P., et al.: SEasonal PErformance factor and MOnitoring for heat pump systems in the building sector SEPEMO-Build: Final report. Intelligent Energy Europe (2012)

[6] Lowe, R., Summerfield, A., Oikonomou, E., Love, J., Biddulph, P., Gleeson, C., Chiu, L.-F., Wingfield, J.: Final report on analysis of heat pump data from the Renewable Heat Premium Payment (RHPP) scheme (2017)

[7] O'Hegarty, R., Kinnane, O., Lennon, D., Colclough, S.: Air-to-water heat pumps: Review and analysis of the performance gap between in-use and product rated performance. Renewable and Sustainable Energy Reviews **155**, 111887 (2022) https://doi.org/10.1016/j.rser.2021.111887

[8] Gleeson, C.P., Lowe, R.: Meta-analysis of European heat pump field trial efficiencies. Energy and Buildings **66**, 637–647 (2013) https://doi.org/10.1016/j.enbuild.2013.07.064

[9] Pospíšil, J., Špiláček, M., Kudela, L.: Potential of predictive control for improvement of seasonal coefficient of performance of air source heat pump in Central European climate zone. Energy **154**, 415–423 (2018) https://doi.org/10.1016/j.energy.2018.04.131

[10] Lämmle, M., Metz, J., Kropp, M., Wapler, J., Oltersdorf, T., Günther, D., Herkel, S., Bongs, C.: Heat pump systems in existing multifamily buildings: A meta-analysis of field measurement data focusing on the relationship of temperature and performance of heat pump systems. Energy Technology, 2300379 (2023) https://doi.org/10.1002/ente.202300379

[11] Ruhnau, O., Hirth, L., Praktiknjo, A.: Time series of heat demand and heat pump efficiency for energy system modeling. Scientific Data **6**(1), 1–10 (2019) https://doi.org/10.1038/s41597-019-0199-y

[12] Fischer, D., Wolf, T., Wapler, J., Hollinger, R., Madani, H.: Model-based flexibility assessment of a residential heat pump pool. Energy **118**, 853–864 (2017) https://doi.org/10.1016/j.energy.2016.10.111
